# Pig manure treatment strategies for mitigating the spread of antibiotic resistance

**DOI:** 10.1038/s41598-023-39204-4

**Published:** 2023-07-25

**Authors:** Magdalena Zalewska, Aleksandra Błażejewska, Agnieszka Czapko, Magdalena Popowska

**Affiliations:** Department of Bacterial Physiology, Faculty of Biology, Institute of Microbiology, University of Warsaw, Warsaw, Poland

**Keywords:** Biotechnology, Microbiology, Molecular biology, Environmental sciences, Natural hazards, Risk factors

## Abstract

Due to the risk of pathogenic antibiotic-resistant bacteria and their antibiotic-resistance genes transfer from livestock feces to the soil and cultivated crops, it is imperative to find effective on-farm manure treatments to minimize that hazardous potential. An introduced worldwide policy of sustainable development, focus on ecological agricultural production, and the circular economy aimed at reducing the use of artificial fertilizers; therefore, such treatment methods should also maximize the fertilization value of animal manure. The two strategies for processing pig manure are proposed in this study—storage and composting. The present study examines the changes in the physicochemical properties of treated manure, in the microbiome, and in the resistome, compared to raw manure. This is the first such comprehensive analysis performed on the same batch of manure. Our results suggest that while none of the processes eliminates the environmental risk, composting results in a faster and more pronounced reduction of mobile genetic elements harboring antibiotic resistance genes, including those responsible for multi-drug resistance. Overall, the composting process can be an efficient strategy for mitigating the spread of antibiotic resistance in the environment and reducing the risk of its transfer to crops and the food chain while providing essential fertilizer ingredients.

## Introduction

The growing demand for meat has led to an increase in pig farming worldwide. Annual global pig production has increased in the last 50 years, reaching approximately 120 million tons in 2018. Total swine meat consumption is estimated to rise by 13% in 2030 and 22% in 2050 compared with 2020^1^. The main pig meat producers are China (45% of global production), followed by the United States, Germany, Spain and Vietnam^2^, with these five countries accounting for nearly 65% of world pig meat production. While in the EU, antibiotics are only applied to treat bacterial infections, in some other countries, they are also commonly applied to promote growth and increase production efficiency; in these countries, approximately 66,667 tons of antibiotics are applied to livestock worldwide each year^3^. However, due to the link reported between antibiotic use in livestock and the emergence of antibiotic resistance in pathogenic bacteria, the use of antibiotics as growth promoters is being limited and has been prohibited in the EU since 2006^4^; it has also been restricted in the United States since 2017^5^ and in China since 2020^6^. Nevertheless, China remains the largest producer and consumer of antibiotics, with 52% being used in animal production^7^.

While the swine gastrointestinal microbiota harbors a diverse population of bacteria known to support the health of the host, they may also be a source of drug resistance genes. Intensive livestock farms are characterized by a combination of high bacterial load and high antimicrobial selection pressure: conditions known to promote the occurrence of antimicrobial-resistant bacteria (ARB) and antibiotic resistance genes (ARG)^8^. In addition, disturbances in gut microbiota may enhance ARG transfer or enhance the abundance of ARB shed from the animal.

Typically, more than half of the administered dose of antibiotics is excreted unchanged in the urine and feces. Only a small number of antibiotics are partially metabolized by the host animal, yielding microbiologically active or inactive metabolites. For example, in the liver, enrofloxacin is partially (< 25%) metabolized to ciprofloxacin, while sulfonamides are metabolized to a small extent to the less active N4-acetylosulfonamides; in both cases, the products are microbiologically active^9^.

As a single pig may produce up to 6.4 kg of wet manure per day^10^, large animal breeding facilities produce vast amounts of animal feces, resulting in the production of 1.7 billion tons of feces annually worldwide^11^. In pig farms in Germany, Spain, the UK and the Netherlands alone, annual manure production amounts to over 120 million tons^12^. The easiest and cheapest way to dispose of such waste is land application; however, livestock manure has been identified as a reservoir of antibiotics, ARGs, and potentially pathogenic ARB, posing a considerable threat to animals and human health. Indeed, pig manure is regarded as a key source of the spread of antibiotic resistance to the environment^13,14^. Certainly, the direct application of livestock manure originating from farm animals treated with antibiotics is known to expose agricultural areas to high levels of antibiotics. Depending on the physio-chemical properties of the antibiotic and soil composition, the compounds may remain in the manure-amended soil, or may be transferred to ground and surface waters by leaching or runoff. Further, pollutants can be carried by water over long distances and affect the entire aquatic ecosystem. In addition, the rate of degradation varies depending on the antibiotic type, and in many cases, this can be very low for up to 30 days^14^. When in soil, the compounds may be taken up by crops, where they accumulate and then enter the food chain. In addition, the constant presence of a low concentration of antibiotics and their active residues in the environment favors the selection of resistant bacteria^14^.

The most commonly-applied manure management strategy is storage, where the feces are simply set aside in a designated secure location for a prolonged time. This requires sufficient storage capacity, as the minimal storage periods before spreading varies from 4 to 7.5 months, depending on the animal type, the length of time at pasture, and the geographical location^15^. Alternatively, manure can also be composted: an environmentally and economically-beneficial practice for processing solid organic waste, allowing for efficient volume reduction, pathogen removal, and antibiotic content reduction^16^. Storage and composting are part of the principles of green chemistry; however, manure composting may raise legitimate environmental concerns^17–19^. Methane is formed as a result of anaerobic decomposing of organic matter. In aerobic conditions methane is oxidized to carbon dioxide, which is less contributing to the global warming than methane. Improperly conducted composting process can lead to increased methane emissions. Agriculture sector contributes most to the anthropogenic methane emission (50,63%), but the emission related to manure management practices consist the smallest part of it (59,84% related to enteric fermentation, followed by emission from rice cultivation, other agricultural practices and manure management) and is still decreasing (measured from 1990 to 2010)^20^. In the proposed study, we focused on pig manure, not pig slurry. Liquid material (pig slurry), when stored in manure pits or lagoons, has a greater probability to develop anaerobic processes and thus develop higher methane production than in case of dry manure^21^. However, it is hard to determine the methane production from management system in advance, it depends on composition of manure, storage/composting conditions (pH, temperature, moisture or C/N content), animal type or even animal diets^20^.

Currently, little data exists on the distribution of ARGs in solid pig manure, and its microbial community structure; in addition, no studies have compared the composting and storage of material obtained from the same source. The aim of the present study is to evaluate the role of pig manure as a crucial hot-spot for the spread of ARGs into the environment, even considering the strict control of antibiotic application for livestock in EU member countries. The findings highlight the need to introduce composting for treating manure before its land application, rather than its storage. It also determines the transmission potential of ARGs by identifying the correlations between microbial phyla and ARG groups, and between ARG and mobile genetic elements (MGE) observed during both storage and composting. To determine how the spread of AMR can best be reduced before manure is applied to the fields, the study compares the effects of two commonly-used manure management strategies, storage and composting, at the laboratory scale. When performing the analyses, we assumed the type of process as the independent variable, and ARG relative abundance and microbial phylum relative abundance as the dependent variables. It is important to note that all samples used in the two methods were collected from the same animals from a single farm and at the same time point. The analysis consists mainly of high-throughput qPCR to determine the changes in ARG distribution across all samples, and sequencing protocols targeting V3-V4 variable regions of 16S rRNA to allow microbial community identification.

## Materials and methods

### Sample collection

The pig manure sample were collected from a Polish commercial finisher farm in Spring (April) 2019. Manure and slurry were separated on-farm. A solid fraction was collected in two blue plastic drums of approximately 20 L each (one for composting and the other for storage), and was taken to the laboratory before further processing and analysis. The farm owner agreed to manure sampling and shared the history of the use of antibiotics on the farm. Specific location of the farm and farmer details are not disclosed due to privacy restrictions. Antibiotics were applied according to EU legislation–Regulation (EU) 2019/61 on Veterinary Medicines and Regulation (EU) 2019/4 on Medicated Feed^22^. The pig herd consists of 200 pigs (50 pigs per group pen), weight gain of 1100 g/day/pig. Diet used: 80% grain meal from own farm, 20% complementary feed: protein base, soybean meal or rapeseed meal + vitamins, micro- and macronutrients.

### Pig manure management strategies and sampling

5 kg of pig manure was mixed with Sitka spruce sawdust at the ratio of 4 (manure):1 (sawdust) (w/w) and placed in a bucket. Sawdust was obtained from the local hardware store. The organic matter, total nitrogen, phosphorus, calcium, magnesium, and heavy metal (Cr, Cu, Cd, Ni, Pb, Zn, Hg) content in raw manure and after storage (4 M) and composting (10W) was determined: Hg content according to the procedure described in DIN ISO 16772, other heavy metals (ICP-OES/ICP-MS)–DIN EN ISO 11885/DIN EN ISO 17294-2, total nitrogen–VDLUFA, Bd. I, Kap. A 2.2.1, ammoniacal nitrogen–DIN 38406 E5-1 mod., dry organic matter–DIN EN 12879. Only Hg and dry organic matter content were determined according to the dedicated accredited norm; other components were analysed according to procedures described in norms but adjusted to our needs.

Although the duration of both processes differs, the composting and storage periods were applied according to commonly used practices—the time of 4 months for storage was set up following the EU legislation, and ten weeks was sufficient time to complete the composting as described by Ros et al.^23^. We aimed to compare commonly recommended manure treatment strategies along with suggested durations.

#### Composting

The container with the mixture was placed in the laboratory under the ventilated hood at ambient temperature. Compost was turned (mixed well) each week for ten weeks. The temperature, moisture, and pH were measured in the compost at about 15 cm (half the mixture's height) each week. The moisture content was measured periodically and adjusted to 50–60% by adding sterile MilliQ water (to avoid potential contamination with ARGs or bacteria). The experiment was conducted in triplicate. Samples for the microbiological and molecular analysis were collected at the beginning, in the middle (fifth week), and at the end of the process (tenth week).

#### Storage

The container with the material was placed in the laboratory under the ventilated hood at ambient temperature; however, the material was not mixed. The temperature, moisture, and pH were measured in the material at about 15 cm (half the height) each week. The experiment was conducted in triplicate. Samples for the microbiological and molecular analysis were collected at the beginning of the process, in the middle (second month), and at the end (the fourth month).

### Sampling strategies

We collected altogether 200 samples in whole experiment. We take 40 samples per group gathered together from three technical repetitions per group (control, composted samples after five weeks and ten weeks and stored samples after two months and four months). Isolated DNA from group was pooled together according to groups and send for analysis.

### DNA extraction and metagenomic sequencing

Total DNA was isolated from 500 mg of the manure sample using FastDNA™ SPIN Kit for Feces (MP Biomedicals) following the manufacturer's recommendations. The quality and quantity of extracted DNA were analysed with an Invitrogen Qubit 4.0 Fluorometer (dsDNA high-sensitivity assay kit) and Colibri spectrophotometer (Titertek Berthold). The DNA samples were isolated in triplicate and then pooled to obtain a single DNA sample for each time point.

The characteristics of the bacterial community structure were determined via 16S rRNA sequencing targeting the variable V3-V4 regions of bacterial 16S rRNA. Following the manufacturer’s recommendations, the libraries were prepared with Nextera® XT index Kit v2 Set A (Illumina). The prepared amplicons were then subjected to high-throughput sequencing using the MiSeq Illumina platform to generate 2 × 300 bp paired-end sequences at the DNA Sequencing and Oligonucleotide Synthesis Facility (Institute of Biochemistry and Biophysics Polish Academy of Sciences). Raw sequences were processed and analysed using Qiime2 software^24^ with the DADA2 option for sequence quality control and the newest release of the SILVA ribosomal RNA sequence database for taxonomy assignment^25,26^. The obtained data was analysed and visualized using MicrobiomeAnalyst^27^.

### High-throughput qPCR and primers

The qPCR array was performed in the SmartChip Real-time PCR system by Resistomap (Finland). The qPCR cycling conditions and initial data processing were performed as described previously^28^. The same 384-well template of primers was used for each DNA sample. Three technical replicates were run for each sample. The samples used for the analysis have similar quality: 260/280 ratio of 1.8–2.0 (+ /−0.1) and a concentration of 10 ng/μl. The concentration of the sample was measured using the Qubit 4.0 Fluorimeter (Thermo Fisher Scientific), and the quality using a Colibri spectrophotometer (Titertek Berthold). Only samples matching the described criteria were analysed. After amplification based on the qPCR SmartChip Real-Time PCR cycler (Takara), threshold cycle (CT) values were calculated using default parameters provided with the SmartChip analysis software; the genes and primer sequences are listed in Supplementary file 4. The gene names and groups listed and categorized in the sheet 1 in Supplementary file 4 are used throughout the manuscript.

Melting curve and qPCR efficiency analysis were performed on all samples for each primer set. Amplicons with unspecific melting curves and multiple slope profile peaks were considered false positives and discarded from the analysis. Samples fitting the following criteria were taken for further analysis: (1) Ct values ≤ 27, (2) at least two replicates, (3) amplification efficiency in the range of 1.8 to 2.2. The relative copy number was calculated using  Eq. (1)^29^. The gene copy numbers were calculated by normalizing the relative copy numbers per 16S rRNA gene copy numbers.

1 $$Relative \,gene \,copy \,number={10}^{(27-Ct)/(10/3)}$$

### Statistical analysis

The minimal sample size was calculated using G*Power software (version 3.1.9.7)^30,31^.

Data obtained from metagenomic sequencing targeting V3-V4 regions of 16S rRNA gene and High-Throughput qPCR were collected in Excel files (Microsoft Office 2019) and are attached in xlsx files as supplementary materials. Calculation of ARG prevalence was done in Excel according to Eq. (1).

The results of bacterial community sequencing and antibacterial resistance genes groups prevalence were analysed and visualized using MicrobiomeAnalyst 2.0 web server^27^. For bacterial community richness and diversity determination, Shannon and Chao1 indexes were used. The differences between abovementioned parameters were determined using Kruskal–Wallis one-way analysis of variance non-parametrical tests. Principal Coordinates Analysis were done using Bray–Curtis dissimilarity index. All tools were provided by MicrobiomeAnalyst tool. The parameters differs significantly when *p* < 0.05.

The relationships between ARG and MGEs and between ARG and microbial taxa were determined using Spearman’s non-parametrical correlation coefficient. The calculation of Spearman’s correlation coefficient and data visualization were performed with GraphPad Prism 9 (Graph Pad Prism version 9.0.0). The analysed relationships were used to construct networks using Cytoscape (Cytoscape version 3.9.0). The correlations were considered strong and significant when the absolute value of Spearman's rank |r|> 0.7 and *p* < 0.05^32^.

## Results

### Physio-chemical properties of pig manure

The organic matter (OM) content was found to be static after storage, but a slightly increase was observed after composting. In addition, both sets of treated matter showed enrichment of N but a reduction in concentration of P, Mg and Ca compared with baseline. The manure pH was found to rise slightly from 7.0 to 7.4 during composting; however, a similar rise was observed during storage. The humidity, pH and temperature were measured during the process in weekly manner for ten weeks. The concentrations of heavy metals remained below the applicable standards in UE considered safe. All results are presented in Tables S1 and S2 (Supplementary file 1).

### Microbial composition of samples

**Figure 1:**
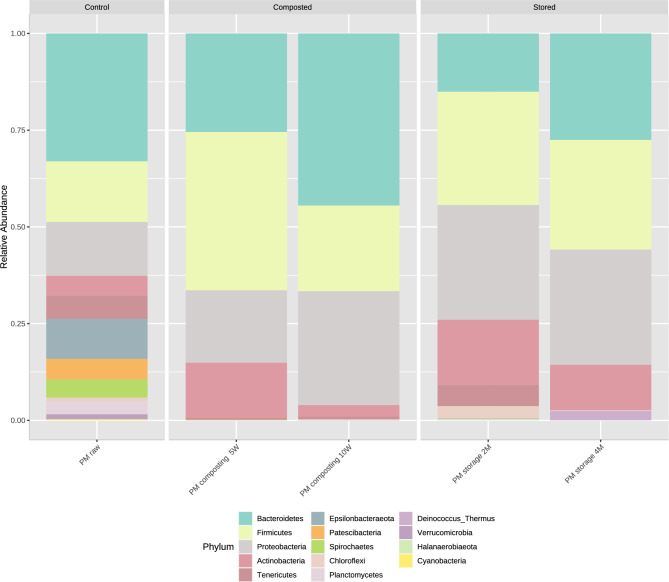
Microbial composition of samples at the phylum level. The samples were divided into three groups–control (PM raw; sample before treatment), composted (PM composting 5W, PM composting 10W; composted samples after five weeks and ten weeks, respectively), and stored (PM stored 2 M, PM stored 4 M; stored samples after two months and four months, respectively); stacked barplots represents average value for three replicates.

The composition of bacterial communities at the phylum level is presented using the relative abundance graph type (Fig. 1). The data are available as supplementary file 2.

The relative abundance of *Bacterioidetes* decreased after five weeks (18.8%) and then increased after ten weeks (43.6%) during composting; however, this value decreased after two months (14.7%) then increased at four months (51.3%) during storage. The relative abundance of *Firmicutes* increased after five weeks (30.2%) and decreased after ten weeks (21.7%) during composting but continuously increased during the storage (28.5% and 52.8, after two and four months, respectively). The relative abundance of *Proteobacteria* increased during both composting (13.8% after five weeks and 28.8% after ten weeks) and storage (28.2% after two months and 55.5% after four months). Finally, the prevalence of *Actinobacteria* initially increased (10.6%) but later decreased at the latest stages (3%) during composting; however, its level increased continuously from 16.4% after two months and 21.8% after four months during storage.

### Microbial community diversity

**Figure 2:**
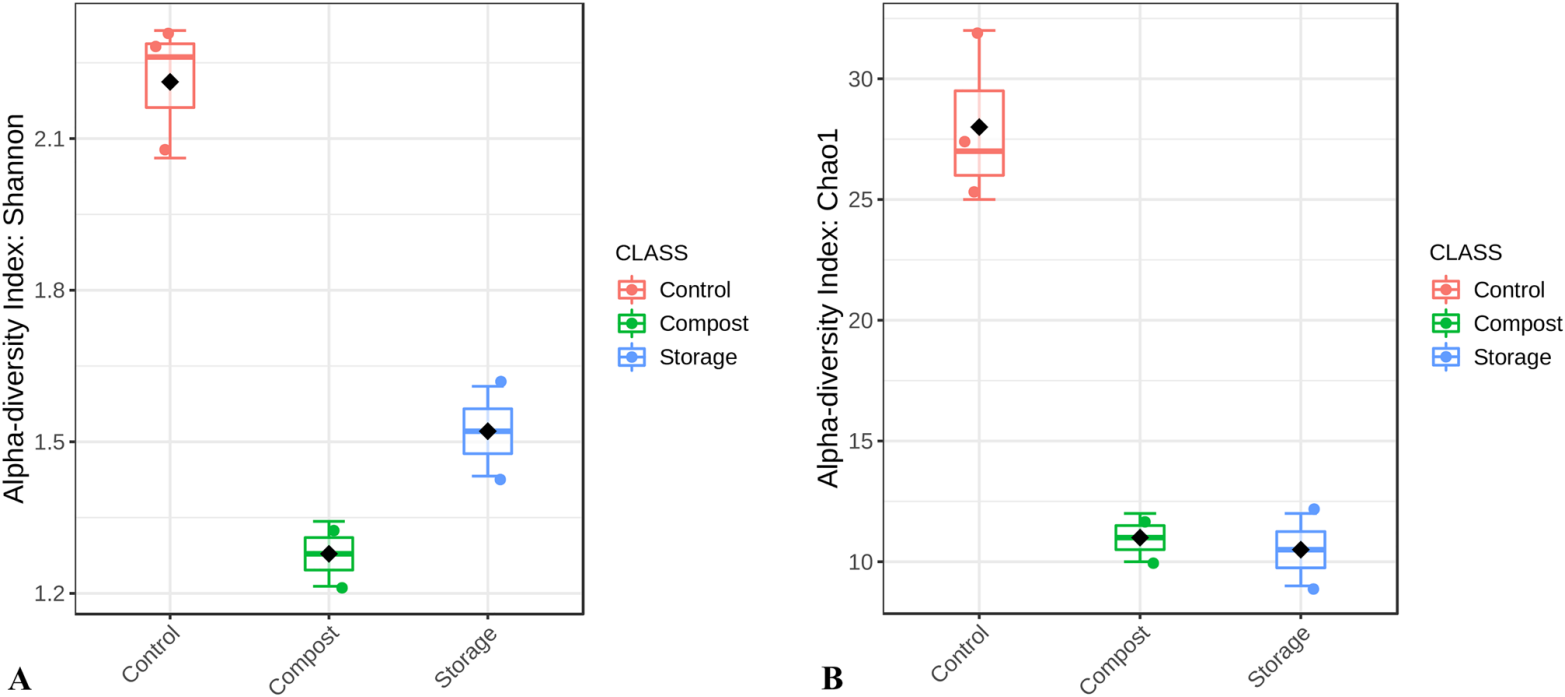
(**A**) Shannon indexes of microbial community diversity (*p *value 0.07 [Kruskal–Wallis]) (**B**) Chao 1 indexes of microbial community richness (*p* value 0.1 [Kruskal–Wallis]); the samples were divided into three groups–control (PM raw 1, PM raw 2, PM raw 3; samples before treatment), composted (PM composting 5W, PM composting 10W; composted samples after five and ten weeks, respectively), and stored (PM stored 2 M, PM stored 4 M; stored samples after two and four months, respectively).

The richness and diversity of the microbial communities were analyzed using the Shannon and Chao1 indexes (Fig. 2). The Shannon index showed lower values during storage and composting than the untreated control samples, but only as a trend. Chao1 index also showed lower values during storage and composting than the untreated control samples at the trend level. A principal coordinate analysis (PCoA) was used to determine the differences between all samples based on the Bray–Curtis dissimilarity index; the PCoA plot did not show any differences between samples depending on management strategies (untreated manure included).

Although Shannon and Chao1 indexes do not differ significantly, the microbial community composition varied between treated and raw pig manure. Many bacterial phyla decline during composting and storage, but none appears (supplementary file 3).

### Diversity and abundance of ARGs

**Figure 3:**
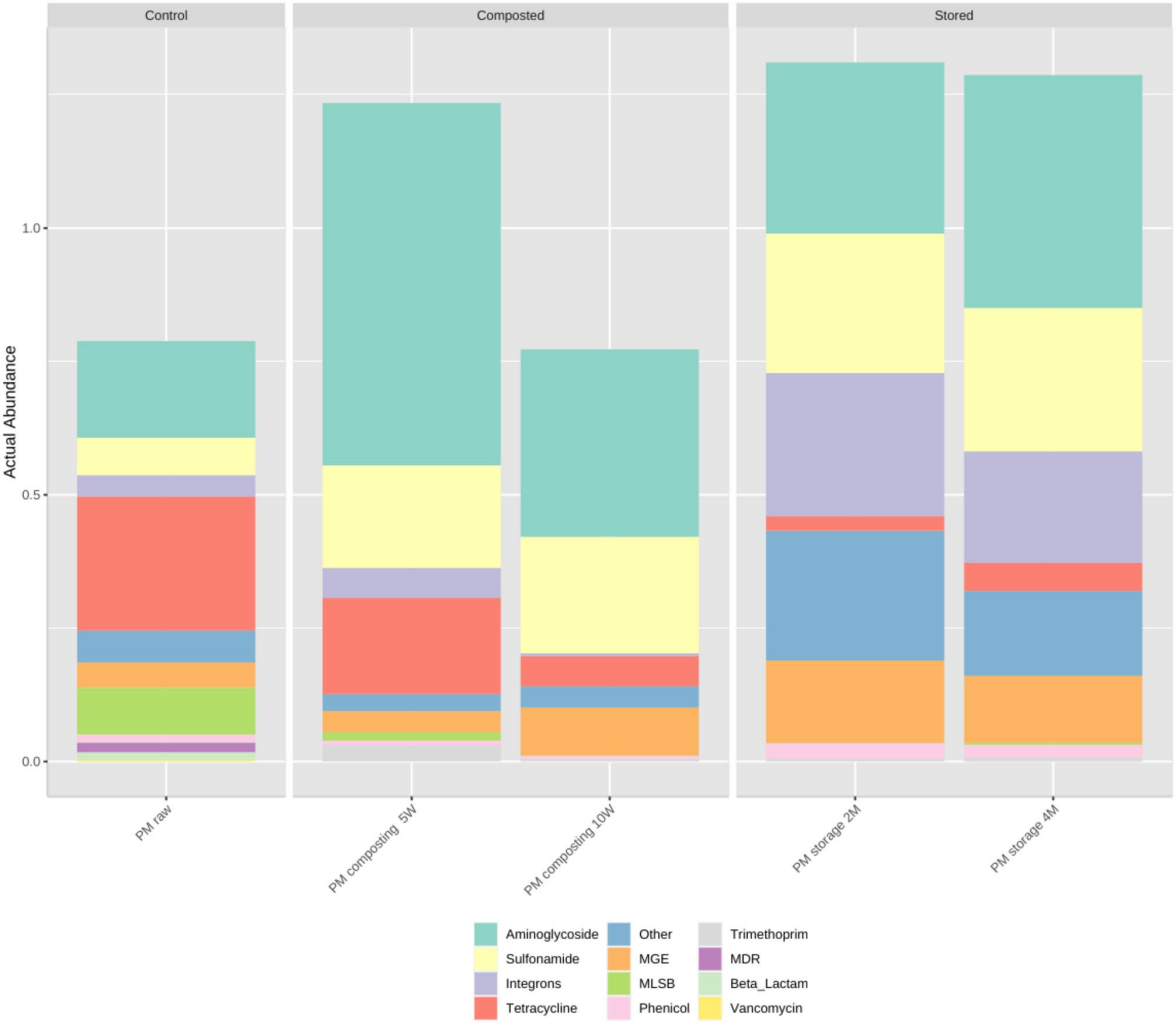
Relative abundances of detected antimicrobial resistance genes and mobile genetic element gene classes detected during qPCR analysis. The samples were divided into three groups–control (PM raw; sample before treatment), composted (PM composting 5W, PM composting 10W; composted samples after five and ten weeks, respectively), and stored (PM stored 2 M, PM stored 4 M; stored samples after two and four months, respectively); stacked barplots represents the average value for three replicates.

The relative abundances of the analyzed gene classes and MGEs varied during composting and storage (Fig. 3) (raw data are available as Supplementary file 4). In both cases, a higher total abundance of detected genes was observed in treated compared to untreated manure; however, the distribution of individual ARGs and ARG groups was found to differ depending on treatment type. The changes in the presence of individual genes are visualized by a heatmap (Supplementary file 5). In raw manure, the highest number of detected genes were associated with tetracycline resistance, followed by aminoglycoside, MLSB, and sulfonamide resistance. The lowest abundances were detected for phenicol-, vancomycin-, and trimethoprim-resistance genes.

Composting causes a temporary increase in the total amount of detected genes to 1.23 gene copies/16S rRNA gene copies after five weeks, followed by a decrease to 0.77 gene copies/16S rRNA gene copies after ten weeks. The composting process caused a decline in the total number of tetracycline-resistance genes; it also resulted in a decrease in the numbers of MLSB-, beta-lactam-, phenicol-, and vancomycin-resistance genes, as well as genes known to offer resistance against other antimicrobials. Composting also caused a reduction in the relative amounts of genes responsible for multi-drug resistance (MDR), as well as in the relative abundance of genes classified as integrons. However, the process resulted in an increase in the total amounts of aminoglycoside-resistance genes and sulfonamide-resistance genes, as well as the amount of MGEs.

In contrast, during storage, the relative abundance of genes increased to 1.31 gene copies/16S rRNA gene copies after two months, and then decreased after four months (1.29 gene copies/16S rRNA gene copies). The storage process caused a decline in tetracycline-, MLSB-, beta-lactam-, and vancomycin-resistance genes, and many aminoglycoside-, sulfonamide-, phenicol-, and trimethoprim-resistance genes were detected after four months of treatment. Storage also caused an increase in the number of resistance genes against other antimicrobials, integrons and MGEs. For both processes, the number of detected genes remained elevated compared to the initial level of 0.79/16S rRNA gene copies. However, in the case of composting, all genes reached their lowest levels after week 10.

While the relative abundance of ARGs was found to generally increase during both treatment strategies this tendency varied depending on the gene class (Table S3, Supplementary file 1). The relative abundance of beta-lactam-, tetracycline-, vancomycin-resistance genes, genes conferring the MLSB resistance phenotype, and genes coding the MDR phenotype were reduced at the end of composting and at the end of storage. Interestingly, while the relative abundance of integrons decreased during composting, their copy number increased during storage. A similar situation was observed with phenicol-resistance genes: their abundance decreased after composting but increased after storage for four months. In addition, resistance genes classified as other decreased after composting and increased after storage. The number of copies of the remaining analyzed ARGs groups increased during both manure management strategies, but the size degree of the change varied according to the process.

**Figure 4:**
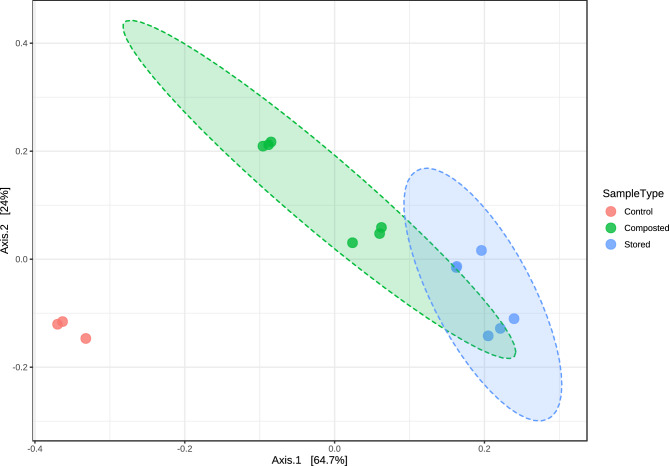
Principal coordinate analysis (PCoA) of ARG and MGE relative abundances in all sample groups based on the Bray–Curtis distance method. The ANOSIM statistical method was used. [ANOSIM] R: 0.98401; *p* value < 0.001. The samples were divided into three groups–control (PM raw 1, PM raw 2; samples before treatment), composted (PM composting 5W, PM composting 10W; composted samples after five and ten weeks, respectively), and stored (PM stored 2 M, PM stored 4 M; stored samples after two and four months, respectively).

The PCoA the relative abundances of ARGs and MGEs in all sample groups, calculated based on the Bray–Curtis distance method, revealed significant visual separation between the initial (untreated) and treated samples with regard to the relative abundances of resistant gene classes (*p* < 0.05) (Fig. 4).

**Figure 5:**
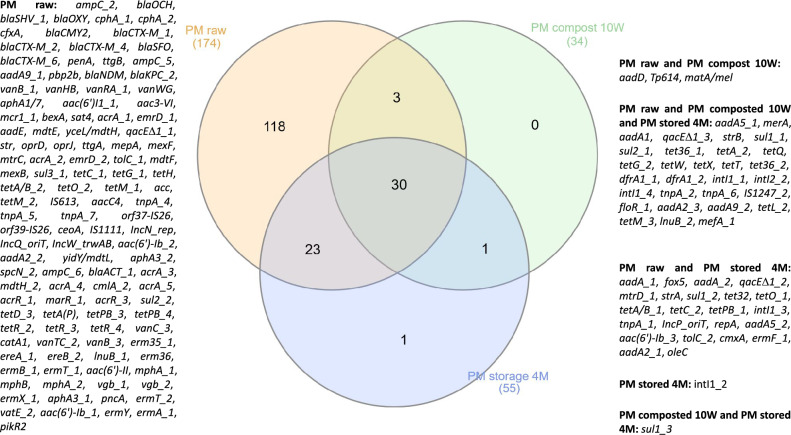
Core resistome in pig manure samples. The Venn diagram, presenting the number of detected genes across all samples and their intersection, was created by InteractiVenn^33^. The samples were divided into three groups: PM raw–untreated pig manure, PM compost 10W–pig manure after 10 weeks of composting, PM storage 4 M–pig manure after four months of storage.

The most prevalent gene in raw pig manure was *aadE*, followed by *tetM_1*. Both genes were effectively reduced during both composting and storage. Of the ten most common genes in raw pig manure (*aadE, tetM_1, tetM_3, tetPB_3, tnpA_2, qacEΔ1_2, tetQ, sul2_2, tetW, tetM_2*–ranked from high to low), two increased after treatment: *tnpA_2*, and *qacEΔ1_2*. The former belongs to the *IS21* group of transposases and was assigned during this study to the MGE group, while the latter codes efflux pumps, which is one of the mechanisms responsible for MDR. Both composting and storage caused a reduction in the abundance of 80% of the genes detected in raw manure; of these, one is an aminoglycoside-resistance gene, six are tetracycline-resistance genes, one was a MGE, one an MDR mechanism, and one a sulfonamide resistance gene. The core resistome of the analyzed samples is presented in Fig. 5.

The applied manure management strategies were found to only partially reduce ARG diversity (Table S4, Supplementary file 1). The number of integrons increased during four-month storage, but decreased during composting. The number of trimethoprim-resistance genes was not reduced during either procedure. The numbers of aminoglycoside-, beta-lactam-, phenicol-, sulfonamide-, tetracycline-, vancomycin-resistance genes, genes coding the MLSB resistance phenotype, genes conferring the MDR phenotype, MGEs, resistance genes against other antimicrobials were decreased.

### Correlation analysis of ARGs, MGEs, and microbial communities across the manure treatment processes in pig manure

**Figure 6:**
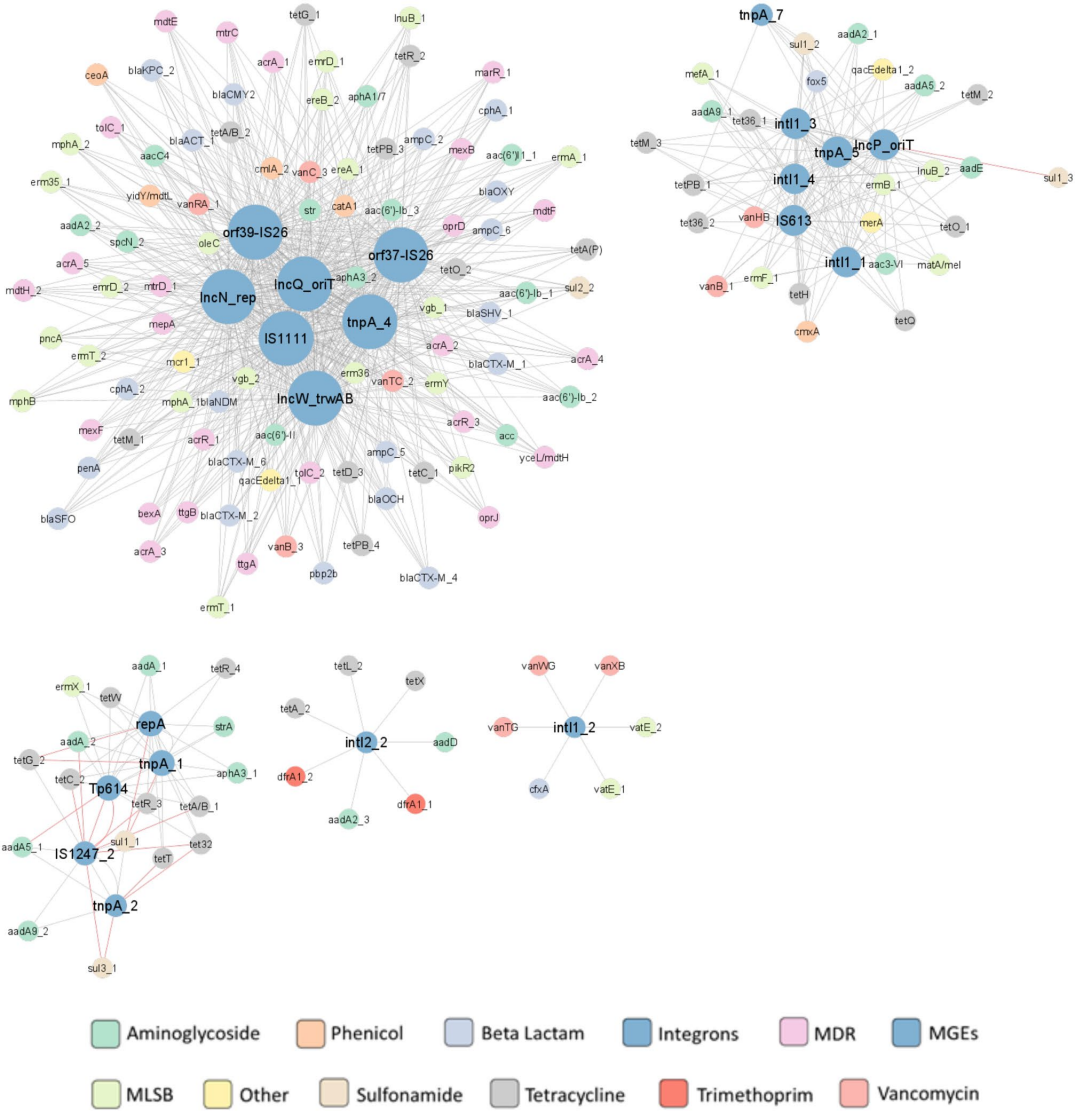
ARG and MGE interaction network in compost. The network is presented as an 'organic layout'. A strong and significant correlation is shown, based on Spearman's rank correlation, where |r|> 0.9 and *p* < 0.05. The size of the nodes represents the degree of interaction. The gray and orange edges show positive and negative correlations between ARGs and MGEs, respectively. The color of the nodes indicated the ARG type according to the legend.

A co-occurrence network was constructed to determine the relationship between 157 ARGs and 21 MGEs (integrons included) during composting. This network (Fig. 6) contains, altogether, 179 nodes, and was created based on 959 strong (|r|> 0,9) and significant correlations (*p* ≤ 0.05) with 942 positive correlations and 17 negative ones. The MGEs showed more connections than ARGs, indicating a more important role in network formulation. Among the MGEs and integrons group*, orf39-IS26, IncN_rep, IncQ_oriT, orf37-IS26, IS1111, tnpA_4, IncW_trwAB* showed the highest number of correlations with ARGs. The rest of results is presented in Fig. 6.

**Figure 7:**
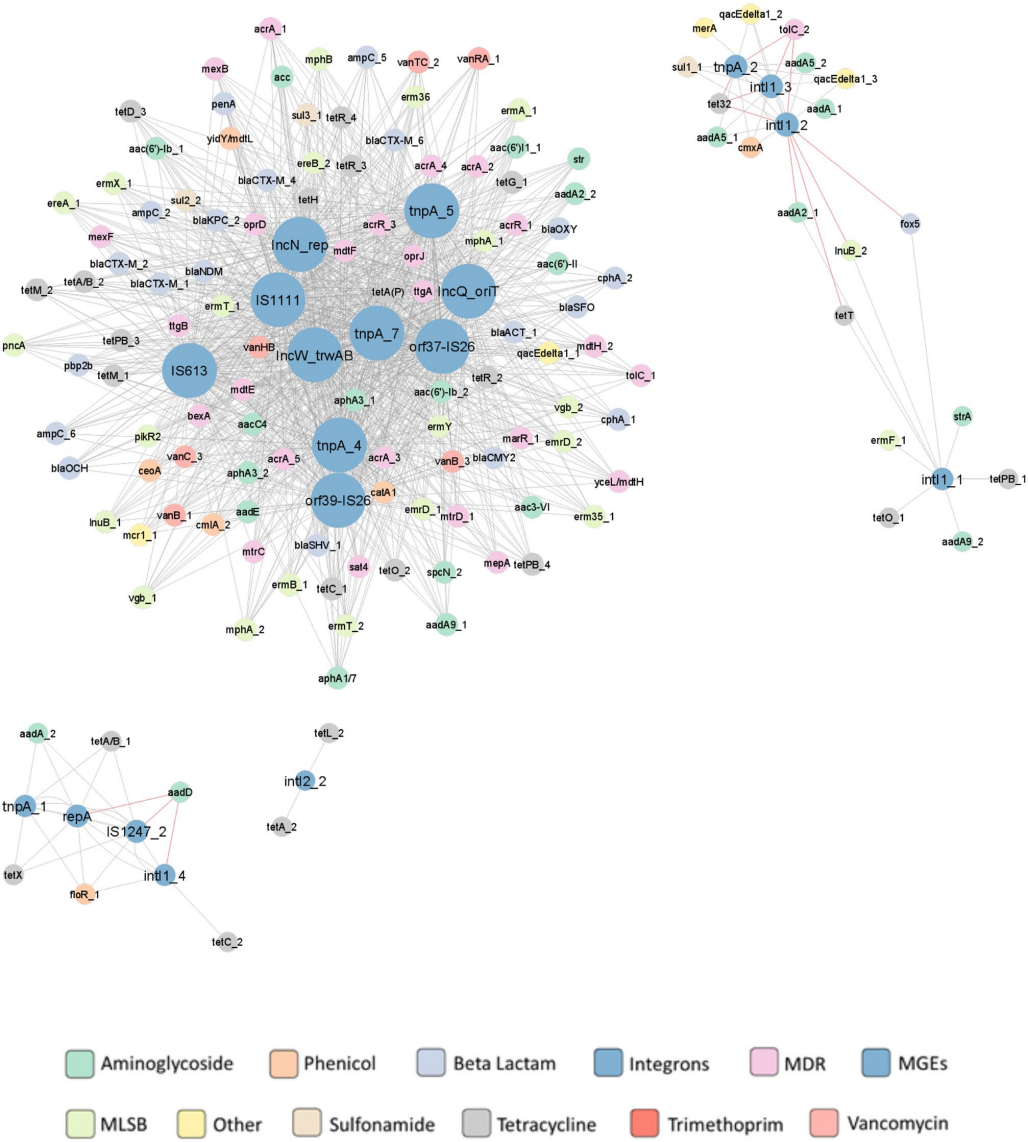
ARG and MGE interaction network in compost. The network is presented as an 'organic layout'. A strong and significant correlation is shown based on Spearman's rank correlation, where |r|> 0.9 and *p* < 0.05. The size of the nodes represents the degree of interaction. The gray and blue edges show positive and negative correlations between ARGs and MGEs, respectively. The color of the nodes indicates the ARG type according to the legend.

The relationship between 155 ARGs and 19 MGEs (together with integrons) during storage was determined by constructing a co-occurrence network. The analysis comprised 155 ARGs and 19 MGEs assessed across the storage process. This network (Fig. 7) contained a total of 152 nodes, and it was created based on 1224 strong (|r|> 0,9) and significant correlations (*p* ≤ 0.05) with 1211 positive correlations and 13 negative ones. The MGEs showed more connections than ARGs, indicating they play an important role in network formulation. Among the MGE and integron group, *orf39-IS26, IncN_rep, IncQ_oriT, orf37-IS26, IS1111, tnpA_4, IncW_trwAB, tnpA_5 tnpA_7, intI1_3, IS613, intI1_4, intI1_1, repA, tnpA_1, IS1247_2, tnpA_2, intI2_2*, showed the highest number of correlations with ARGs. The rest of results is presented in Fig. 7.

### Correlation between detected genes in HT-qPCR and microbial taxa

**Figure 8:**
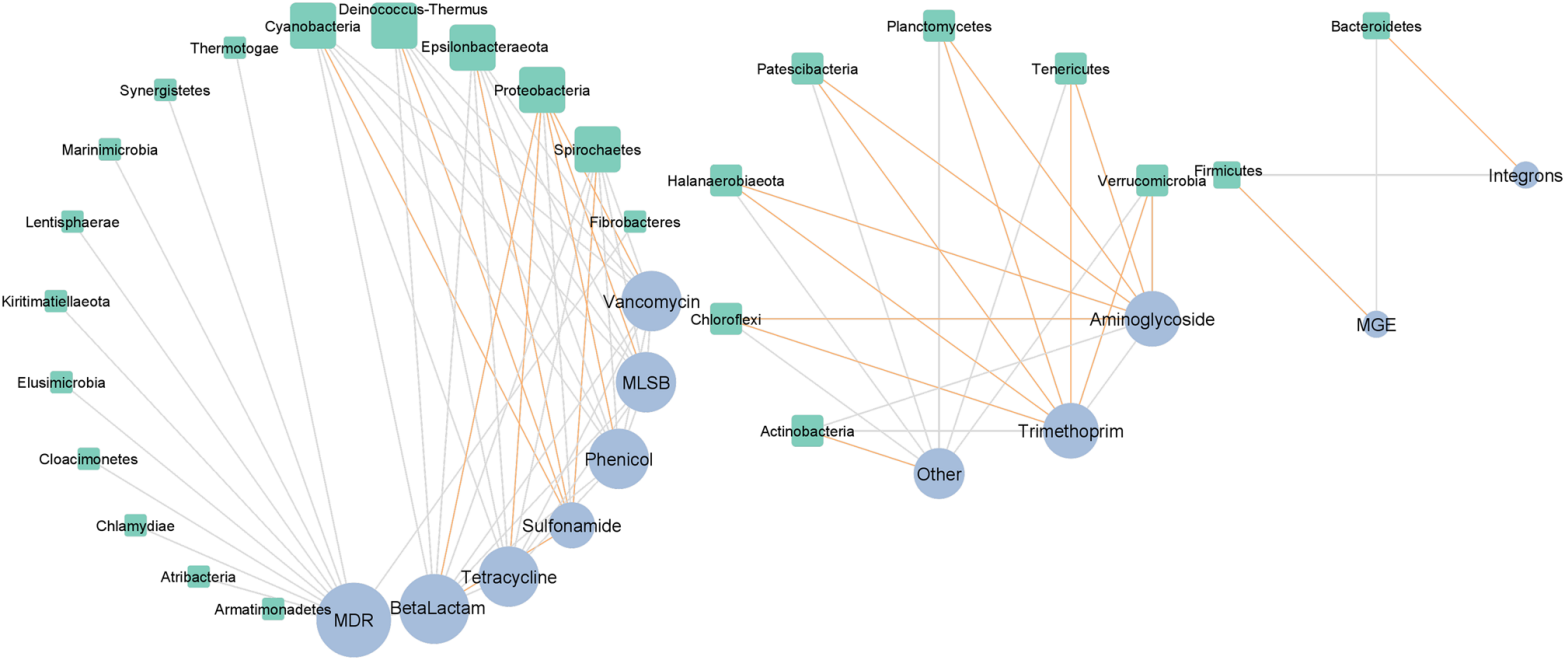
Interaction network analysis of microbial taxa and gene classes during composting, presented as a 'circular layout' based on the co-occurrence of ARG groups and microbial phyla. A connection is significantly correlated with Spearman's |r|> 0.85 and *p* < 0.05. The gray and orange edges represent the positive and negative correlations between ARG groups and phyla, respectively. The size of the nodes shows the degree of interaction. Green nodes represent microbial phyla, and blue nodes represent groups of ARGs.

The interactions between 26 phyla and 12 gene classes (10 ARGs classes, integrons, and MGEs) identified during the composting procedure were investigated. The resulting co-occurrence network (Fig. 8) contained 37 nodes, and it was created based on 79 strong (|r|> 0,85) and significant (*p* ≤ 0.05) correlations with 54 positive and 25 negative correlations. As shown in the network, the gene classes presented more interactions than the microbial phyla, indicating that these genes play a crucial role in the co-occurrence network construction. While the genes coding the MDR mechanism demonstrated the highest interaction with microbial phyla, genes coding the resistance to aminoglycosides, trimethoprim, and genes assigned as Other class also exhibited high levels of interaction. The lowest level of connections were observed for integrons and MGEs. The rest of results is presented in Fig. 8.

**Figure 9:**
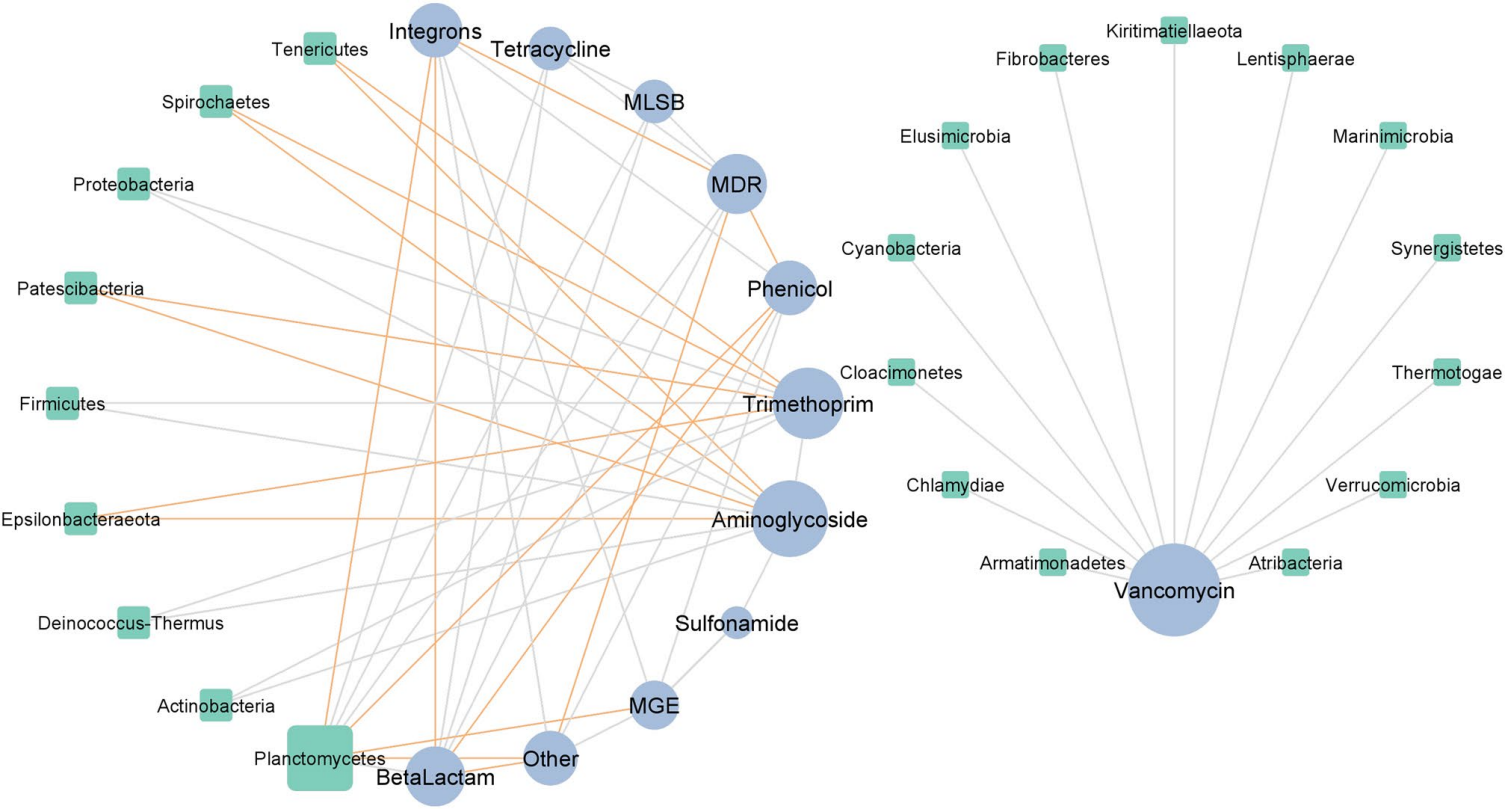
Interaction network analysis of microbial taxa and gene classes during storage, presented as a 'circular layout' based on the co-occurrence of ARG groups and microbial phylum. A connection is significantly correlated when Spearman's |r|> 0.85 and *p* < 0.05. The gray and orange edges represent the positive and negative correlations between ARG groups and phyla, respectively. The size of the nodes shows the degree of interaction. Green nodes represent microbial phyla, and blue nodes represent groups of ARGs.

The interactions between 26 phyla and 12 gene classes (10 ARG classes, integrons and MGEs) identified during pig manure storage were investigated. The constructed co-occurrence network (Fig. 9) contained 34 nodes, and was created based on 58 strong (|r|> 0.85) and significant (*p* ≤ 0.05) correlations: 40 positive and 18 negative correlations. Apart from *Planctomycetes*, all bacterial phyla demonstrated either negative or positive correlations with the studied gene classes. Of the phyla, *Planctomycetes* demonstrated the most correlations, i.e. three positives (MDR, MLSB, and tetracycline-resistance genes) and four negatives (MGE, integrons, genes classified as other-, and phenicol-resistance genes). The rest of results is presented in Fig. 9.

## Discussion

### Manure treatment

The long-term goal of manure management is to remove environmental contaminants: disposing of the high volume of waste, using its value to improve soil fertility, and ensuring biosecurity. Hence, it was necessary to study the physicochemical properties of both treated and untreated manure.

Composted pig manure has an alkaline pH, which contributes to maintaining or increasing the pH of the soil; this is of great importance for maintaining the proper nitrogen cycle and phosphorus availability, and increasing the absorption of micronutrients by plants^34,35^. Using composted manure to increase the organic matter of soils has an agronomic justification and is also consistent with the EU’s circular economy action plan^36^: it improves the physical (soil structure) and physicochemical conditions of the soil by increasing its sorption properties (the role of humus) and organic substance content; this stimulates the soil microbiome, which in turn triggers a further chain of changes beneficial for plants^37^.

To halt the spread of ARGs and potentially pathogenic microorganisms from animal feces to arable fields and then to crops, various manure pre-treatment strategies have been explored. An important goal in this regard is to identify practices that can decrease their concentrations rather than simply diluting them^38^. The abundance of ARGs in livestock animal manure after composting is highly variable. It depends on several essential factors, including the reproduction and death rates of the intestinal microorganisms carrying ARGs, the pressure placed on them by antibiotic residues and heavy metals, favoring the persistence of resistance genes in the bacterial community, and the possibility of horizontal gene transfer between bacteria^39^.

When quantifying the antibiotic resistome, it is important to consider that changes in resistance gene abundances may simply be due to changes in underlying microbial structure, since community composition can also influence the composition of resistance genes^40^. In the present study, the bacterial community underwent changes during composting and storage; however, no significant differences were noted in bacterial community richness or differences, with only general trends observed in microbial community diversity and richness. The most prevalent phyla observed across all samples were *Firmicutes, Bacteroidetes*, and *Proteobacteria*. These three phyla have been found to be the most abundant in the gut microbiome of pigs^41^. In both cases, *Bacteroidetes* was observed to dominate in raw manure, with its numbers falling in the middle of the treatment, then rising towards the end; however, while composting resulted in its final level being higher than initial levels, storage only returned this to initial levels. For *Proteobacteria* and *Actinobacteria*, the numbers increased in the middle of both processes; however, the number of *Actinobacteria* declined at the end of composting. *Firmicutes* has a high tolerance to high temperatures, and hence is more abundant during the thermophilic phase, while *Bacteroidetes* prefers lower temperatures and is more abundant during the cooling phase^42^. Similar results were obtained by Do et al.^43^, but in liquid pig slurry rather than solid manure; however, other researchers have also confirmed these observations for solid pig manure^44–46^. Although storage is the most commonly applied manure management technology, few studies have examined its effect on the bacterial community. *Firmicutes, Actinobacteria*, and *Bacteroidetes* have been found to be the most prevalent bacterial phyla in pig manure during storage under diverse conditions^47^, which is in agreement with our present findings. Do et al.^43^ also report that these phyla dominate in stored manure at different time points.

The network analysis of bacterial phyla and ARG groups in the composted manure found *Proteobacteria* to co-occur with sulfonamide-resistance genes, *Actinobacteria* with aminoglycoside-resistance genes, *Bacteroidetes* with MGEs, and *Firmicutes* with integrons. Our results partially agree with those of a co-occurrence analysis by Liu et. al.^42^, who find that all bacterial phyla (*Proteobacteria, Bacteroidetes, Firmicutes*, and *Actinobacteria*) that predominated during composting are strongly associated with tetracycline-resistance genes, quinolone-resistance genes, and sulfonamide-resistance genes. In addition, Song et al.^48^ also found these phyla to be associated with macrolide-resistance genes. A similar analysis performed on manure sampled during storage found *Actinobacteria, Firmicutes* and *Proteobacteria* to be associated with that of trimethoprim-resistance genes and aminoglycoside-resistance genes. However, those are only calculated based on Spearman’s correlations and they need to be evaluated by experimentation: other research teams may set different parameters or cut-off points.

Although composting is efficient at antibiotic removal (approximately 64.7% of detectable veterinary antibiotics were efficiently removed from manure during composting)^49^ the situations regarding ARG removal is more complicated. Our study detected an elevated ARG and MGE abundance after all applied manure treatment strategies; however, the smallest increase was observed for samples collected after 10 weeks of composting. Similar increases in resistance gene prevalence has been previously noted by Cao et al.^50^ and recently by Do et al.^43^. Moreover, in the PCoA plot of ARGs, the cluster assigned to the initial untreated samples was clearly separate from those assigned to treated manure; however, the two clusters belonging to stored and composted samples overlap. Despite this, the efficiency of ARG and MGE removal is not always satisfactory: some studies report an increase in their relative abundances, while others report a decrease^51^. A study on swine manure composting yielded the following removal rates for genes divided according to antibiotic groups^13^: beta lactam 36.8–73.7%, FCA 11.8–52.9%, MLSB 0–47.6%, tetracycline 4.5–36.4%, vancomycin 0–71.4%, aminoglycoside 0–26.9% and sulfonamide 0%. In our present study, composting resulted in reductions for beta-lactam- (100%), vancomycin- (100%), MLSB- (86.36%), phenicol- (83.33%), aminoglycoside- (79.31%), other (71.43%), tetracycline- (65.52%), and sulfonamide-resistance genes (40%), with no reduction observed for trimethoprim-resistance genes. These results only partially agree with ours. Although we found an increase in MGE abundance, as reported previously^43^, this increase was smaller after composting than after storage.

The core resistome determining the resistance genes which are not removed by any applied treatment, was also identified in the present study. These genes harbor resistance to tetracyclines (*tet36, tetA, tetQ, tetG, tetW, tetX, tetT, tetL, tetM*), aminoglycosides (*aadA5, aadA1, strB, aadA2, aadA9*), sulfonamides (*sul1, sul2*), trimethoprim (*dfrA1*), florfenicol (*floR*), antibiotics from MLSB group (*lnuB, mefA*) heavy metals (*merA*), as well as MGEs (*IntI1, intI2, tnpA, tnpA, IS1247*), and genes encoding the MDR phenotype (*qacEdelta1*). All of the listed genes harbor resistance to antibiotic groups commonly used in both veterinary and medical treatments^52^.

It is known that the correct development of composting depends on its active and curing phase. Pezzola et al.^53^ demonstrated that the maximum attainable temperature, the time to reach it, and the pH and moisture depend on the used waste type and bulking agents. In addition, the dynamics of antibiotics removal appear to be closely influenced by the choice of bulking agent (sawdust rice husk, mushroom residues)^49^.

Although these studies conclude that composting conditions affect the dynamics of antibiotic and ARG elimination from manure; no study has attempted to optimize the process. Studies have found treatment time and temperature to be the key factors affecting changes in ARG level during sludge treatment^54,55^.

Studies on the factors determining ARG profiles during composting have found that certain physicochemical parameters, e.g., temperature, pH, ammonia content, and moisture, have a stronger effect on ARGs than the presence of host bacteria^56–58^. While composting is a dynamic process influenced by the combined activity of a variety of bacteria, actinobacteria and fungi, the dynamics of the bacterial population are directly influenced by the environmental conditions including temperature, pH or moisture; for example, if a thermophilic species of bacteria carries the ARGs, their abundance may increase during the thermal phase^59^.

### Potential for horizontal gene transfer of ARGs

Horizontal gene transfer between different species has been recognized as a common and significant evolutionary process^60^. It is most acutely demonstrated in the close interconnection between the resistome of manure-dwelling gut bacteria and potential pathogens, under constant selective pressure of the antimicrobial substances present in feces. The main vessels of gene flow in microbial communities are plasmids which link distinct genetic pools^61^. Our present findings indicate the presence of plasmids with a broad host range in both raw manure samples and in manure treated with composting or storage. Broad host range plasmids can replicate and stably maintain their gene payloads in taxonomically-distant species.

Our findings indicate the presence of plasmids from the Q, P, N, and W incompatibility groups (*IncQ, IncP, IncN, and IncW*). *IncP* plasmids may transfer between nearly all species of the *Alpha-, Beta-* and *Gammaproteobacteria*, and replicate in them. *IncQ* plasmids are broad host range plasmids that can proliferate in almost all Gram-negative bacteria. In addition, *IncW* and *IncN* plasmids can transfer and replicate in Gram-negative bacteria^62^. The presence of mobile broad host range plasmids plays a key role in the dissemination of beneficial genetic traits, including resistance to antibiotics, metals, quaternary ammonium compounds and triphenylmethane dyes, and degradation of herbicides, both within the population of a certain species, and outside it^14,62^.

Our study also identified *IncP_oriT* and *IncQ_oriT*; *oriT* is the origin of replication in Gram-negative bacteria transfer systems, and is crucial for transferring DNA from the donor to the recipient. Our samples were also found to include the *repA* gene, which encoded the initiation replication protein, ssDNA-dependent ATPase and DNA helicase, which most probably correlated with plasmid-copy number^63^, an internal gene necessary for transposition (*tnpA*), which is necessary for the recognition, cleavage, and integration of transposable elements^64^, and a few insertion sequences (IS): *IS614* (harboring ARGs, e.g., the *cfiA* gene)^65,66^, *IS1247* (encoding MDR phenotype–*aac(6')IIc/ereA2/acc/arr/ereA2*)^67^ and *IS1111* (harboring ARGs–*blaGES-9*)^68^.

Our study compared the abundance of ARGs and MGEs in pig manure, both untreated and after storage or composting. The emergence and spread of ARGs are undoubtfully related to the presence of MGEs such as plasmids, transposases and integrases. A co-occurrence network constructed based on the ARGs and MGEs found in our samples confirmed the co-occurrence of MGEs, integrons and ARGs during both composting and storage. Although the co-occurrence network for the manure treated with storage has fewer nodes than that constructed during composting, it included more positive correlations between MGEs and ARGs, and these also tended to be more centralized: the largest cluster in the storage group includes 10 MGEs, compared to 7 MGEs in composting. Our findings clearly show that even during composting or storing, animal manure should be considered a hot spot for antibiotic resistance in which MGEs and ARGs are present together. Animal manure is a nutrient-rich environment inhabited by a highly-dense bacterial population, which with the addition of antibiotics and heavy metals, makes favorable conditions for horizontal gene transfer. It has been proven that the presence of antibiotics in manure increases the activity of transposases, thus resulting in more frequent excision or integration of gene cassettes in integrons^69^.

The *tet* genes, which were found to be the most prevalent genes in the untreated pig manure, are often identified as plasmid components. Their spread is further facilitated by insertion into transposons such as *Tn6298, Tn1721, Tn6303*, or *IS1216*. For example, the *tetW, tetQ, tetQ* genes are known to be common in the host gastrointestinal microbiota, indicating that the genes demonstrate high stability within the population^69,70^. To date, three sulfonamide-resistance genes have been identified: *sul1*, frequently found within class 1 integrons, *sul2*, found on a broad-host-range plasmid in *E. coli*, and *sul3*, also found together with class 1 integrons in a variety of environments. Due to their location on MGEs, together with integrons and transposons, sulfonamide-resistance genes have high transfer potential and can be found in many bacterial species^39^.

Pig manure is also relatively abundant in beta-lactam resistance genes, since they commonly receive large levels of antibiotics belonging to these groups. The genes are often located within IncN plasmids^71^. IncN plasmids may carry *blaCTX-M*, suggesting that they play a crucial role in the dissemination of ESBL strains in manure. Moreover, the genes coding for the ESBL phenotype are often located on ISEcp1 or ISCR1^72^. Macrolide-resistance genes (e.g., *ermB*) and tetracycline-resistance genes may also be located on MGE, such as the Tn916-Tn1545 family of conjugative transposons. Other interesting genes also have been found in manure associated with MGEs, e.g., the colistin-resistance gene has been found on diverse plasmid replicon types, such as IncX4, IncI, IncFII, IncX1, and IncQ1, in *E. coli*^39^. Other reports have noted the presence of *ampC, qnr*, carbapenemase-resistance genes originating from animal manure on plasmids with a broad host range, such as replicon type plasmids (IncP-1, IncQ, IncN, incW, or IncF), underlining their high transfer potential^73^.

## Conclusions

Our findings reveal an alarming diversity and abundance of ARGs, indicating that raw pig manure represents a ubiquitous reservoir of ARGs and MGEs, which poses a considerable risk of ARGs spreading into the environment when released. The co-occurrence of AGRs and MGEs increases the risk of transfer of ARGs from bacteria inhabiting livestock animals and their environments to human-associated bacterial pathogens, resulting in further spread among human populations. The presence of such resistant strains limits the choice of possible treatment strategies in infection.

Generally, positive correlations were observed between microbial population composition and the presence of specific ARGs and MGEs and genes coding the MDR mechanism, and between ARGs and MGEs in all tested systems. It was found that composting and storage resulted in differential changes in the quantities of ARG groups, while others demonstrated lower, or even higher, gene copy numbers; however, MGEs were found to be one of the groups whose abundance increased during both processes.

The co-occurrence network encompassing both MGEs and ARGs imply that horizontal transfer of genes may occur not only in raw manure, but also during composting or storage before field application. Although the relative abundance of MGEs generally increases during both treatments and their prevalence is associated with a high diversity of resistance genes, a lower overall abundance of MGEs is observed during composting than during storage; this suggests that although composting may not be sufficient to completely limit the spread of ARGs, it is more efficient than simple storage.

Hence, it can be concluded that the use of composted manure is a safer strategy to ensure soil fertility and high humus content. It represents a promising alternative to mineral fertilizers, thus contributing to a more ecological way of farming crops.

## Supplementary Information

Supplementary Information.

## Data Availability

The datasets supporting the conclusions of this article are included within the article and as supplementary materials or, if not, are available from the corresponding author on reasonable request.
