# Supplementary material for: Pig manure treatment strategies for mitigating the spread of antibiotic resistance
Source: Sci Rep. 2023 Jul 25;13:11999. doi: 10.1038/s41598-023-39204-4 (PMC10368742; doi:10.1038/s41598-023-39204-4)
Supplement: Supplementary file 1 — Supplementary Information. [file 41598_2023_39204_MOESM1_ESM.zip › 41598_2023_39204_MOESM1_ESM/Suplementary files information.docx]

Pig manure treatment strategies for mitigating the spread of antibiotic resistance

**Magdalena Zalewska^1^, Aleksandra Błażejewska^1^, Agnieszka Czapko^1^, Magdalena Popowska^1*^**

^1^Department of Bacterial Physiology, Institute of Microbiology, Faculty of Biology, University of Warsaw, Poland

*** Correspondence:** [ma.popowska@uw.edu.pl](mailto:ma.popowska@uw.edu.pl)

Supplementary file 1

Table S1. Physicochemical parameters of pig manure: raw and after composting and storage

Table S2. Reduction [%] of the relative abundances of ARG groups during composting (PM composting 5W, PM composting 10W; composted samples after five weeks and ten weeks, respectively), and stored (PM stored 2M, PM stored 4M; stored samples after two months and four months)

Table S3. Reduction [%] in ARG level during composting and storage (PM composting 5W, PM composting 10W; composted samples after five weeks and ten weeks, respectively), and stored (PM stored 2M, PM stored 4M; stored samples after two months and four months)

Supplementary file 2

Sheet 1. Microbial diversity at the phylum level

The abundance is presented as an average value from three replicates

Supplementary file 3

Venn diagram

Comparison between microbial phyla between untreated and treated pig manure; untreated pig manure [PM raw]: Acidobacteria, BRC1, Chlamydiae, Chrysiogenetes, Cloacimonetes, Cyanobacteria, Deferribacteres, Epsilonbacteraeota, FBP, Fibrobacteres, Gemmatimonadetes, Hydrogenedentes, Kiritimatiellaeota, Lentisphaerae, Spirochaetes, Synergistetes; [PM raw] and pig manure composting after 10 weeks [PM compost 10W]: Patescibacteria, Verrucomicrobia; [PM raw] and [PM compost 10W] and pig manure storage after 4 months [PM storage 4M]: Actinobacteria, Bacteroidetes, Chloroflexi, Firmicutes, Halanaerobiaeota, Planctomycetes, Proteobacteria, Tenericutes; [PM raw] and [PM storage 4M]: Deinococcus-Thermus

Supplementary file 4

Sheet 1. Gene relative abundances

Analyzes genes groups, names, functional classifications, primer sequences, relative abundances

Sheet 2. No. of genes

Number of detected genes classified into gene classes

Sheet 3. Reduction in ARG diversity

The reduction in ARG numbers during treatments classified into gene classes

Sheet 4. Reduction if ARG copies

The reduction in ARG copies during treatment classified into gene classes

Supplementary file 5

Changes in detected ARG and MGE relative abundances depending on manure treatment strategies compared with control. The samples were divided into three groups – control (PM raw, sample before treatment), composted (PM composting 5W, PM composting 10W; composted samples after five and ten weeks, respectively), and stored (PM stored 2M, PM stored 4M; stored samples after two and four months, respectively); heatmap represents average value for three replicates
