# Supplementary material for: Pig manure treatment strategies for mitigating the spread of antibiotic resistance
Source: Sci Rep. 2023 Jul 25;13:11999. doi: 10.1038/s41598-023-39204-4 (PMC10368742; doi:10.1038/s41598-023-39204-4)
Supplement: Supplementary file 1 — Supplementary Information. [file 41598_2023_39204_MOESM1_ESM.zip › 41598_2023_39204_MOESM1_ESM/Supplementary file 1 - 16.06.2023.docx]

**Table S1**. Physicochemical parameters of pig manure: raw and after composting and storage

| **Parameters** | **Sample type** | | |  |
| --- | --- | --- | --- | --- |
|  | **Control** | **Storage (4M)** | **Compost (10W)** |  |
| **pH** | 7.0 | 7.2 | 7.4 |  |
| **DM**  **(% of w.t.)** | 27.63±1.28 | 32±1.02 | 54.25±0.11 |  |
| **OM**  **(% of d.m.)** | 89.60±5.17 | 91.45±1.47 | 98.01±0.89 |  |
| **Manurial**  **[% d.m.]** |  | | |  |
| **(N-NH_4_^+^)** | 1.25±0.09 | 1.18±0.10 | 2.13±0.12 |  |
| **Total N** | 3.11±0.23 | 6.12±0.31 | 6.85±0.09 |  |
| **Total P** | 1.55±0.15 | 1.14±0.08 | 0.81±0.02 |  |
| **Total Mg** | 1.28±0.11 | 1.12±0.07 | 0.88±0.02 |  |
| **Total Ca** | 3.62±0.31 | 2.86±0.26 | 2.32±0.08 |  |
| **Heavy metals**  **(mg/kg)** |  | | | **Permissible standard for agricultural land (mg/kg)***** |
| **Chrome (Cr)** | 4.37±0.31 | 4.42±0.24 | 4,65±0.29 | 150-500 |
| **Copper (Cu)** | 128±9.00 | 146±8.00 | 215±11.00 | 100-300 |
| **Cadmium (Cd)** | 0.561±0.02 | 0.612±0.02 | 0.624±0.01 | 2-5 |
| **Nickel (Ni)** | 6.41±0.56 | 6.88±0.85 | 7.39±0.48 | 100-300 |
| **Lead (Pb)** | 2.49±0.17 | 2.52±0.11 | 2.68±0.12 | 100-500 |
| **Zinc (Zn)** | 796±15.00 | 809±14.00 | 816±13.00 | 300-1000 |
| **Mercury (Hg)** | 0.0468±0.01 | 0.0532±0.01 | 0.0571±0.01 | 2-5 |

*Values ​​depending on the type/properties of arable soil by applicable standards in Poland

DM – dry matter; OM – organic matter

Dry matter (DM) of pig manure was assessed by oven drying of a known wet weight of sample at 105 °C for 24 hours. The dry matter content was determined by the following formula:

DM (%) = [Dry sample weight/Wet sample weight] x 100

The organic matter (OM) was determined by ignition of the dry weight at 550 °C for 2 hours and measuring the weight of the ash. The OM was calculated by the equation:

OM (%) =[(Dry sample weight−ash sample weight)/Dry sample weight] x 100

**Table S2:** Humidity (0-10 points), pH and temperature changes during manure treatments

|  | 3 days | 1 week | 2 weeks | 3 weeks | 4 weeks | 5 weeks | 6 weeks | 10 weeks |
| --- | --- | --- | --- | --- | --- | --- | --- | --- |
|  | Humidity [0-10] | | | | | | | |
| Compost | 6.5 | 6.0 | 6.0 | 5.5 | 5.5 | 5.0 | 4.5 | 4.0 |
| Storage | 7.5 | 7.0 | 7.0 | 7.4 | 8.0 | >8 | >8 | >8 |
|  | pH | | | | | | | |
| Compost | 7.0 | 6.5 | 6.8 | 7.2 | 7.0 | 7.4 | 7.4 | 7.4 |
| Storage | 6.2 | 5.6 | 6.2 | 6.4 | 6.6 | 6.6 | 6.4 | 6.4 |
|  | Temperature [ °C] | | | | | | | |
| Compost | 23.0 | 32.5 | 38.5 | 42.8 | 48.5 | 38.0 | 27.5 | 25.0 |
| Storage | 21.4 | 22.8 | 23.2 | 22.4 | 21.4 | 20.9 | 20.4 | 21.0 |

**Table S3**. Reduction [%] of the relative abundances of ARG groups during composting (PM composting 5W, PM composting 10W; composted samples after five weeks and ten weeks, respectively), and stored (PM stored 2M, PM stored 4M; stored samples after two months and four months)

| ARGs group | PM composting 5W | PM composting 10W | PM storage 2M | PM storage 4M |
| --- | --- | --- | --- | --- |
| Aminoglycoside | -274.91 | -94.15 | -77.22 | -141.12 |
| Beta Lactam | 98.03 | 100 | 100 | 99.63 |
| Integrons | -40.20 | 86.76 | -565.95 | -418.24 |
| MDR | 100 | 100 | 100 | 99.08 |
| MGE | 15.80 | -91.34 | -228.13 | -167.30 |
| MLSB | 82.22 | 99.12 | 99.20 | 95.97 |
| Other | 46.93 | 33.39 | -307.30 | -166.60 |
| Phenicol | 35.31 | 59.00 | -112.66 | -73.88 |
| Sulfonamide | -173.86 | -210.85 | -272.20 | -283.04 |
| Tetracycline | 28.20 | 77.42 | 89.18 | 79.07 |
| Trimethoprim | -1867.09 | -205.23 | -239.12 | -380.87 |
| Vancomycin | 95.7 | 100 | 100 | 100 |

**Table S4.** Reduction [%] in ARG level during composting and storage (PM composting 5W, PM composting 10W; composted samples after five weeks and ten weeks, respectively), and stored (PM stored 2M, PM stored 4M; stored samples after two months and four months)

| ARGs group | PM composting 5W | PM composting 10W | PM storage 2M | PM storage 4M |
| --- | --- | --- | --- | --- |
| Aminoglycoside | 48.28 | 79.31 | 68.97 | 62.07 |
| Beta Lactam | 85.00 | 100.00 | 100.00 | 95.00 |
| Integrons | -25.00 | 25.00 | -25.00 | -25.00 |
| MGE | 47.06 | 78.43 | 64.71 | 64.71 |
| MDR | 100.00 | 100.00 | 100.00 | 96.00 |
| MLSB | 63.64 | 86.36 | 86.36 | 81.82 |
| Other | 42.86 | 71.43 | 57.14 | 57.14 |
| Phenicol | 66.67 | 83.33 | 66.67 | 66.67 |
| Sulfonamide | 0.00 | 40.00 | 20.00 | 20.00 |
| Tetracycline | 34.48 | 65.52 | 65.62 | 48.28 |
| Trimethoprim | 0.00 | 0.00 | 0.00 | 0.00 |
| Vancomycin | 20.00 | 100.00 | 100.00 | 0.00 |
