# Supplementary material for: Pig manure treatment strategies for mitigating the spread of antibiotic resistance
Source: Sci Rep. 2023 Jul 25;13:11999. doi: 10.1038/s41598-023-39204-4 (PMC10368742; doi:10.1038/s41598-023-39204-4)
Supplement: Supplementary file 1 — Supplementary Information. [file 41598_2023_39204_MOESM1_ESM.zip › 41598_2023_39204_MOESM1_ESM/Supplementary file 3.pdf]

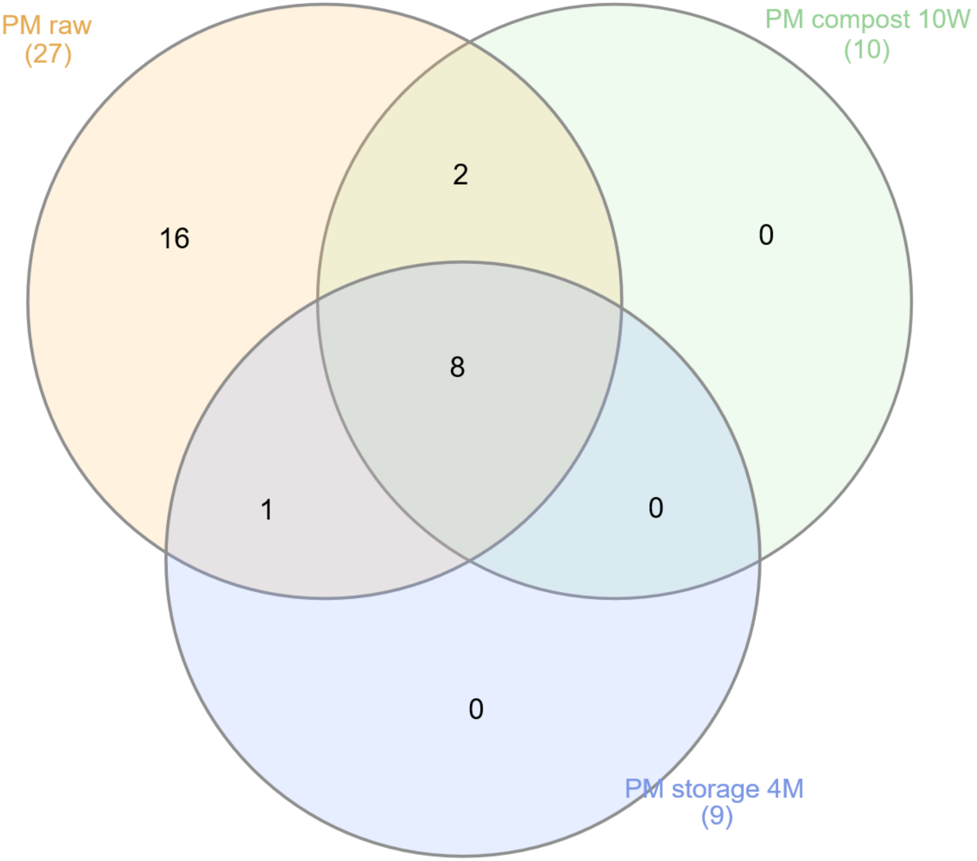

Comparison between microbial phyla between untreated and treated pig manure; untreated pig manure [PM raw]: *Acidobacteria, BRC1, Chlamydiae, Chrysiogenetes, Cloacimonetes, Cyanobacteria, Deferribacteres, Epsilonbacteraeota, FBP, Fibrobacteres, Gemmatimonadetes, Hydrogenedentes, Kiritimatiellaeota, Lentisphaerae, Spirochaetes, Synergistetes;* [PM raw] and pig manure composting after 10 weeks [PM compost 10W]: *Patescibacteria, Verrucomicrobia;* [PM raw] and [PM compost 10W] and pig manure storage after 4 months [PM storage 4M]: *Actinobacteria, Bacteroidetes, Chloroflexi, Firmicutes, Halanaerobiaeota, Planctomycetes, Proteobacteria, Tenericutes*; [PM raw] and [PM storage 4M]: *Deinococcus-Thermus*
