# Supplementary figures and images for: Pig manure treatment strategies for mitigating the spread of antibiotic resistance
Source: Sci Rep. 2023 Jul 25;13:11999. doi: 10.1038/s41598-023-39204-4 (PMC10368742; doi:10.1038/s41598-023-39204-4)

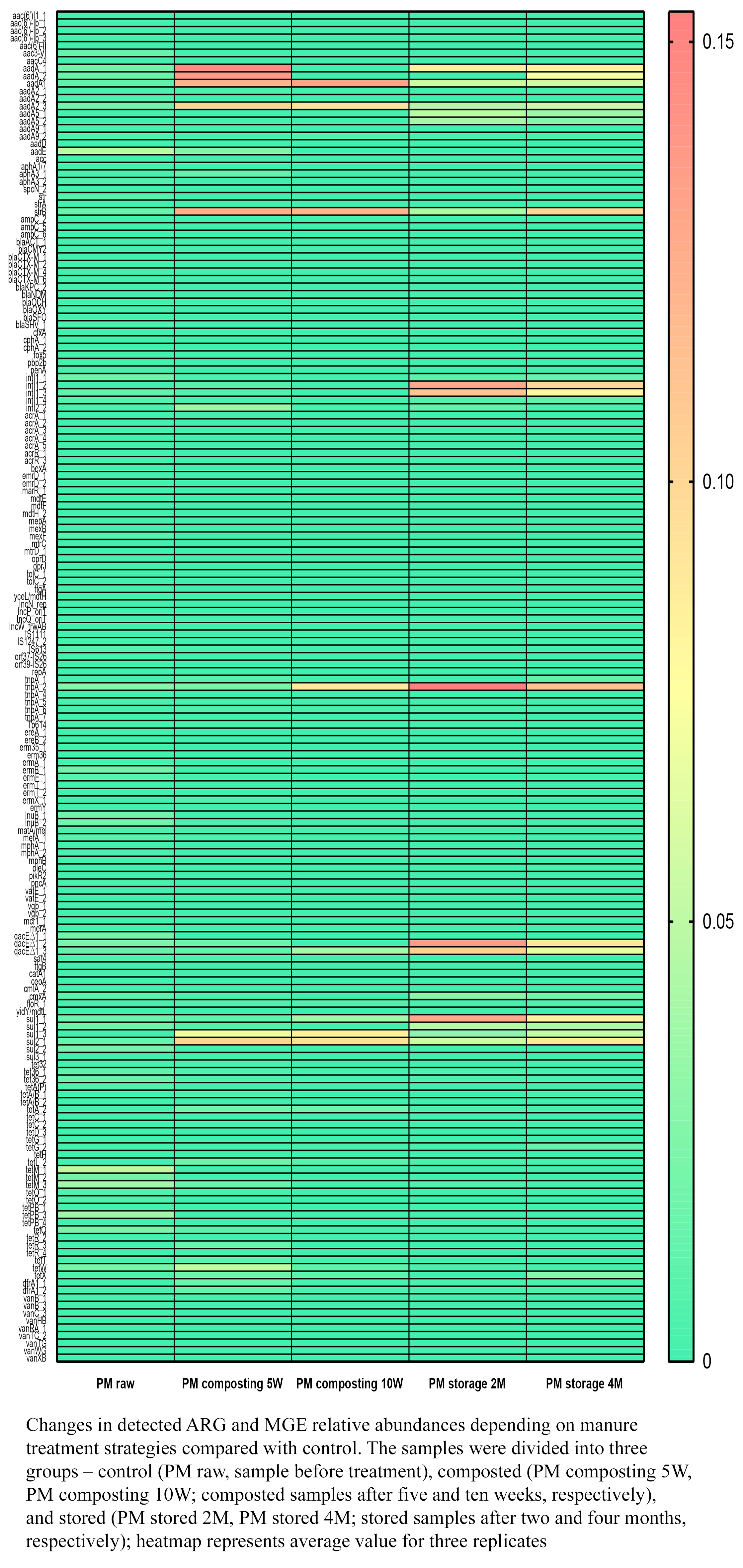

Supplement: Supplementary file 1 — Supplementary Information. [file 41598_2023_39204_MOESM1_ESM.zip › 41598_2023_39204_MOESM1_ESM/Supplementary file 5.tif]
